# Antibodies and Immunity During Kawasaki Disease

**DOI:** 10.3389/fcvm.2020.00094

**Published:** 2020-05-28

**Authors:** Mark Daniel Hicar

**Affiliations:** ^1^University at Buffalo, Buffalo, NY, United States; ^2^John R. Oishei Children's Hospital, Buffalo, NY, United States; ^3^Department of Pediatrics, Jacobs School of Medicine and Biomedical Sciences, University at Buffalo, Buffalo, NY, United States

**Keywords:** kawasaki disease, antibodies-monoclonal, B cells, plasmablast, immunoglobulin intravenous

## Abstract

The cause of Kawasaki disease (KD), the leading cause of acquired heart disease in children, is currently unknown. Epidemiology studies support that an infectious disease is involved in at least starting the inflammatory cascade set off during KD. Clues from epidemiology support that humoral immunity can have a protective effect. However, the role of the immune system, particularly of B cells and antibodies, in pathogenesis of KD is still unclear. Intravenous immunoglobulin (IVIG) and other therapies targeted at modulating inflammation can prevent development of coronary aneurysms. A number of autoantibody responses have been reported in children with KD and antibodies have been generated from aneurysmal plasma cell infiltrates. Recent reports show that children with KD have similar plasmablast responses as other children with infectious diseases, further supporting an infectious starting point. As ongoing studies are attempting to identify the etiology of KD through study of antibody responses, we sought to review the role of humoral immunity in KD pathogenesis, treatment, and recovery.

## Introduction

The cause of Kawasaki disease (KD) continues to perplex clinicians and researchers. Also known as Kawasaki syndrome or mucocutaneous lymph node syndrome, KD is the leading cause of acquired cardiac disease in children. Recent murine and human clinical trials are enhancing our understanding of this disorder (1). The mainstay of treatment has been intravenous immunoglobulin (IVIG), initially implying a major role for humoral immunity. However, newer therapies that also have broad immunomodulatory effects have become widely used for refractory cases (2). Studies from different fields, from maternal immunity to genome-wide association studies, also imply a role of humoral immunity (3–6). This review will give an overview of the present knowledge of the field directed toward the etiology of KD and what role B cells and antibodies may play in treatment, pathogenesis, and diagnosis.

## Review With a Focus on B Cells and Antibodies

### Clinical Presentation

Diagnosis is purely clinical, as there are no adequately specific or sensitive tests available. The “classic” diagnosis involves 5 days of fever and having four of the five following criteria: mucous membrane inflammation, rash, hands and feet swelling, conjunctivitis, and a solitary inflamed lymph node mass (6–9). If left untreated, roughly one-quarter of the children meeting clinical criteria will go on to have coronary artery inflammation, including aneurysms. Incomplete cases, those which do not fulfill four of five of the classic criteria, have similar risk of coronary aneurysms (10). Treating affected patients with IVIG reduces the rates of coronary aneurysms, with a minority seemingly resistant to treatment (6–8, 11–14). Although most aneurysms resolve, some defects are retained. Initial studies done on adults with a history of KD implies there is a greater lifetime risk of cardiac issues and early mortality (15–18). To add to the diagnostic confusion, several infectious etiologies have also been independently associated with aneurysms (19). It remains a frustrating diagnosis because of the unknown etiology, clinical variability, lack of specific testing, and unclear pathogenesis.

### Epidemiology

There appears to be a genetic influence in exhibiting KD. Incidence is higher in some genetic backgrounds and consistently appears in males greater than females within those backgrounds (20). By age five in the United States, 1 in 1,000 African-American children and 1 in 2,000 Caucasian children will have been affected (21–23). In general, Asians have a much higher rate of KD; which is especially evident in Japanese children, whose lifetime incidence rate is near 1% (24). This predisposition holds even for those persons of Japanese heritage raised in foreign lands, such as the United States (20). Sibling can have a 10–30-fold higher risk of KD compared to the general population (25).

The etiology of KD is unknown (6, 26, 27). However, there is a proposed relation to an infectious agent. Epidemiological evidence for this comes from the fact that there are seasonal peaks of KD during winter and spring months and outbreaks have been described (27–34). Siblings have a 10–30-fold higher rate, with most occurring within 1 year of each other (35), and up to 50% of sibling cases are within 10 days of each other (25).

Recent studies support a protective role for humoral immunity. There is a lower incidence in breastfed infants (4) and KD is rare in both newborns and individuals over 5 years of age. This implies a maternally derived protective immunity to a ubiquitous infectious agent (36). Trans-placental passage of maternal antibodies is thought to be protective and explain the paucity of cases in infancy (3).

### Proposed Etiologies

It is possible that there is not one cause of KD, but multiple etiologies that result in similar pathogenesis. This may explain the clinical variability and lack of discovery of a definitive agent, however, the low recurrence rate even in high prevalent areas speaks against a large number of infectious causes (37).

Previously proposed infectious agents include Herpesviridae (HHV-6, Epstein Barr Virus, Cytomegalovirus), human coronavirus, retroviruses, Parvovirus B19, bocavirus, and bacterial infections such as staphylococci, streptococci, *Bartonella*, and *Yersinia* infections (15, 20). Some of these agents have been independently associated with aneurysm formation (19), with the Epstein Barr Virus most commonly associated (38). Several non-infectious agents have also been proposed such as carpet shampoos, mercury exposure and living near bodies of water (15, 20). Additionally, the recent report of tropospheric wind patterns correlating with outbreaks in Japan would not be consistent with many of the viruses that have been proposed (26, 34, 39). These reports imply a relationship to an environmental antigen, as either a priming or inciting event. This “two-hit” hypothesis is also suggested by similar data from Canada (40).

If a ubiquitous childhood pathogen is the cause of KD, the mode of entry would likely be a common mode of infection such as fecal-oral or respiratory spread. Outbreaks in the United States have been associated with preceding viral illness (41). To note, mild upper respiratory symptoms and gastrointestinal complaints have been described in up to 35 and 61% of cases, respectively (42). Rare but more significant pulmonary disease has also been reported (43). Notably, however, concomitant respiratory viruses are near 10% of cases (44, 45). A persistent infection has been theorized (46). Although numerous viruses that can reactivate during stress (Herpesviridae family) or are considered “slow” viral infections (47), the failure of numerous attempts to identify a specific infectious agent argues against a prolonged infection. There are difficult to culture viruses, such as coronavirus which had also enjoyed a short-lived consideration as the cause of KD (48). An abnormal response to normal flora has been proposed (49, 50) and studies on a relationship to the emerging field of microbiome research have recently been reviewed (51).

### Human Biomarkers

Currently, diagnosis is aided by utilizing sensitive but not specific biomarkers such as C-reactive protein, sedimentation rate, liver function tests, urine leukocytes, platelets, leukocyte count, and hemoglobin (2). As highlighted by recommendations for diagnosis of incomplete cases, many biomarkers do not reveal the nature of the underlying illness. A number of traditional laboratory and clinical findings have been built into scoring systems to predict IVIG resistance that are used in Japanese populations (52). These scoring systems (*Sano, Kobayashi, Egami*) all have relatively high sensitivity, near 80%, in Asian populations; however, they have poor predictive ability in heterogenous lower pretest probability populations of North America (53).

Numerous other biomarkers have been proposed to aid in diagnosis of KD and have been recently reviewed (54). Although some of the transcriptomic approaches mirror genomic findings, intriguingly, there has been a disconnect between proteomics and genomic associations. A recent study using serum mass spectrometry to look at differentially expressed proteins showed lipopolysaccharide binding protein (LBP), leucine rich alpha-2 glycoprotein (LRG1), and angiotensinogen were higher in acute phase, and retinol binding protein was diminished (55). The marker of tenascin, involved in tissue remodeling, is promising as a marker of coronary involvement during acute KD (56, 57).

Numerous cytokine associations in KD have been shown, and those with unique B cell associations will be reviewed. IL-10 has long been shown to be elevated during acute KD (58, 59). This is likely a natural anti-inflammatory response, as recent studies show supplementing IL-10 via an AAV vector protected against aneurysms in the *Candida albicans* murine model (60). IL-10 is produced by myeloid dendritic cells and regulatory B cells, and recently has been shown to drive plasmablast responses (discussed later) (61). IP-10, an activator of B cells and macrophages, has also been associated with clinical KD. Notably, this group did not see peripheral IL-1B elevation. (62). IL-21, produced mainly by T cells and Natural Killer cells (63, 64), has recently been proposed as a specific marker in KD in a Korean cohort of children when compared to prolonged fevers from mononucleosis (65). IL-21 modulates immunoglobulin isotype switching and is involved in the differentiation of both naïve and memory B cells into mature plasma cells (66). However, in a study of IL-21 levels in children presenting to a North American emergency room with fever, KD and febrile children could not be distinguished by IL-21 levels (67).

#### Biomarkers Supporting Innate Immunity

A number of transcriptomic approaches show some promise in distinguishing KD from viral infections. Initial studies that look at IVIG response in PBMCs and monocytes suggested monocyte regulation was a main role of IVIG (68) FCGR1a, FCGR3A, CCR2, S100A9, S100A12, and adrenomedullin were notably effected. FCGR2A transcripts were reduced, but surface expression on monocytes was variable. The S100A9 and S100A12 are involved in monocyte adhesion and chemotaxis. Adrenomedullin, important for vascular integrity, was shown in monocytes by gene array as well (69) and these were both supported in other studies (70). Transcriptome analysis of PBMCs showed upregulation of NAIP, IPAF, S100A9, FCGR1A, and GCA which is also supportive of a role for the innate immune system (71).

#### Transcriptomics Supportive of a Role for B Cells in KD

Pathway significance analysis of blood lymphocyte-specific gene markers revealed that PI3K signaling in B lymphocytes was the most significant finding; however, T cell receptor signaling, B cell receptor signaling, T helper cell differentiation, and natural killer cell signaling were also significantly down-regulated in KD compared to febrile control patients (72). On whole blood expression analysis, comparing acute vs. convalescent samples, numerous upregulated pathways involved in innate immunity (particularly IL-1 and the Nlrp3 inflammasome) were shown in the acute phase. IL-10 was notably the highest association, and will be reviewed later. For pathways downregulated, outside of EIF2/p70S6K/MTOR pathways, “B cell development” was the most significant. As mTOR is essential in signaling through PI3K/Akt activity after B cell receptor (BCR) engagement, this association also supports that B cells may be playing a role. A number of these associations; however, were also seen in their controls. There was a surprising similarity with influenza and virally infected individuals. Of the pathways completely unique to KD, most of the specific associations related to myofibroblast migration (paxillin signaling, G-protein coupled receptor signaling, triacylglycerol and relaxin signaling) but PI3K/AKT signaling pathway showed a minor association. Lastly, there was surprisingly few transcript differences noted in those with and without aneurysms (73). A recent similar study comparing KD to adenovirus did not show much specific overlap with previous studies, but was also consistent with relation to a number of inflammatory pathways (74).

Overall, transcriptomics and cytokine profiling suggest a significant, but not exclusive, role for B cells in the pathogenesis of KD.

### Models Systems of KD (See Table 1)

The first KD model system developed depended on intraperitoneal *Candida albicans* alkaline extract injections in susceptible mouse strains (75). Injection of the water soluble fraction of this, (CAWS) had increased incidence of arteritis which can be partially blocked by IVIG. IL-1 inhibition also diminishes the coronary inflammation (76). Notably this vasculitis is a panvasculitis predominantly and also effects the aortic root, which has not been described in KD cases (77).

**Table 1:** Comparison of human KD to common murine models of KD.

	**Human KD**	**CAWS**	**LCWE**
Pathogenesis	Unknown	Superantigen	Superantigen
Etiology	Unknown	*Candida albicans* water soluble injection	*Lactobacillus casei* cell wall extract injection
Animal	Human	Mice	Mice
Arteritis	medium-sized, muscular arteries, includes epicardial coronary arteries	Targets elastic arteries, including aortic root	Elastic arteries, aortitis, proximal coronary arteritis, abdominal aorta dilatations.
Histology	Early neutrophils, mixed data on granulomas	Monocytes, macrophages, neutrophils	Granulomatous
Myocarditis	Subclinical, Tachycardia	Significant	Significant, CK-MB and troponin rise, late myofibrosis
Timing	Adventia/intima to pan vasculitis	Progresses from initial intima layer slowly to panvasculitis	np
Therapy that reduces coronary inflammation	IVIG shows efficacy, Cyclosporine A efficacious in higher risk individuals	TNF a blocks arteritis IVIG partially blocks arteritis IVIG timing influences effect	Diminished by IVIG, anti-TNFα, anti-IL-1
Long term findings	Unclear associations Limited pathologic samples, mixed reports	np	Atherosclerosis, myofibrosis

*np, not published*.

Similarly, mice develop coronary artery inflammation after intraperitoneal injection with *Lactobacillus casei* cell wall extract (LCWE) (78, 79). Pathogenesis in this model parallels KD in that younger mice are more predisposed to develop arteritis and there is a favorable response to IVIG treatment. This disease exhibits mostly a T-cell infiltrate in coronary arterial specimens (79). In fact, in both RAG-1 (80) and TCR-α (81) deficient mice, this arteritis is diminished (82). Etanercept completely blocked these lesions and this was apparently related to signaling through TNFRI (83). Blocking IL-1 can also prevent progression of coronary pathology (84, 85). TNFα can drive metalloproteinase mmp9 activity to cause elastin breakdown. Doxycycline has been shown to prevent this (86), and human data will come from the currently open trial (87). A number of studies show marked myocarditis in these models with late fibrosis (88); however, following troponins is generally not clinically relevant. One group did associate serum troponins and ck-mb (89) but others have not seen that association. A number of the model systems have granulomatous changes, which have variably been seen in human specimens (90, 91). Other models depend on immune complex deposition. This was observed after bovine serum albumin injection into rabbits, which exhibited a disease similar to serum sickness (92). Presently, there is not a model system consistent with direct infectious coronary artery invasion.

No model system exactly replicates the pathologic changes seen in humans, and the utility of these models have been called into question (93). Although most data from model systems are supportive of superantigen involvement, studies from human peripheral lymphocyte responses as reviewed are variable and inconsistent (94). These models and other data are driving clinical trials, but results seen in the models aren't equitably transferable to treatment modalities (reviewed later). Since the cause in humans is unknown, it is still unclear if any of these models of arteritis are truly applicable.

### The Superantigen Theory

A superantigen response was considered by numerous groups and is supported by murine models (79, 95–99). Certain bacterial infections contain proteins that non-specifically bind effector cell receptors causing a more generalized polyclonal expansion and inflammation, termed a superantigen effect. Polyclonality of T cell receptor usage has been shown in KD (100, 101); however, the reports are variable as to which subset of T cell receptors are effected (102). Other studies support a traditional oligoclonal response consistent with an immune response against a specific etiologic agent. Oligoclonal expansion of CD8+ T cells (103) and peripheral IgM+ B cell responses have been shown (9, 104). Numerous other studies have not shown superantigen associated expansions of cell subsets (103, 105, 106). Overall, there is not a clear role for a superantigen response during KD.

### Genetic Implications

Inflammation during KD is universally present, so it is no surprise that a recent network and pathway analysis was consistent with global activation of the immune response (107). Large dataset and related human genomic studies have been performed to look for associated genes with KD incidence and treatment response. These study co-segregation of single nucleotide polymorphisms (SNPs) with the disease state to define significance. These are excellent hypothesis generating techniques, as each dataset generated has numerous implicated genes, but assigning biological significance can at times be difficult. These have been more successful in more homogenous populations and, for KD, have been recently reviewed (108–110).

Genome wide association studies (GWAS) offer unbiased approaches to explore genetic associations. A SNP (rs28493229) near inositol 1, 4, 5-trisphosphate 3-kinase (ITPKC, 19q13) was implicated first by a linkage disequilibrium study in siblings (111). ITPKC modifies the IP3 pathway leading to activation of NFAT transcription factor. A number of GWAS studies were negative or needed metanalysis to confirm this finding (112, 113) or showed a different SNP (rs2290692) association in the area (114). This may be an effect of differing genetic backgrounds in these studies. Other studies associate this gene area with coronary artery risk (115) and a number of SNPs associated with ITPKC were also seen in a study that initially identified FCRG2a (116). Caspase 3 (rs113420705) has been proposed to act downstream of ITPKC, and a genetic variance in the enhancer has been associated (rs72689236) (117, 118). Single-nucleotide polymorphisms (SNPs) in ITPKC (rs28493229) and caspase-3 (CASP3, rs113420705), when analyzed together, associate with increased risk for aneurysms (119). Separately they show a trend of overrepresentation in IVIG non-responders and together with coronary risk (120). These two genes could work on a similar calcium influx pathway. The polymorphism rs28493229 has been shown to effect ITPKC protein levels and thereby IL-1β and inflammasome NLRP3 expression, which was shown by studying murine models, KD subject samples, and EBV transformed B cells (121). There is a general lack of studies in non-Asian populations and disparate results can be seen depending on the genetic background. One of the first studies of this nature showed NAALADL2 and ZFHX3 highly associated with susceptibility in Dutch Caucasians. Reanalysis implicated other genes as well (DGKB, PPP1R14C, LNX1, CAMK2D, CSMD1, and TCP1) and network analysis could associate a number of these with CAMK2D (122).

The most significant and repeatedly associated susceptibility genes are FCGR2A, BLK and CD40, as reviewed in (110). The rs1801274 SNP is a functional polymorphism associated in the IgG receptor gene FCGR2A, which is a cell surface receptor found on phagocytic cells such as macrophages and neutrophils (116, 123). This receptor is a low-affinity receptor for immunoglobulin and is involved in the process of phagocytosis and clearing of immune complexes. Intriguingly, male gender influences the association of the FCRG2a (124). Genome-wide genetic marker association studies show that specific polymorphisms in CD40 and in the B lymphoid tyrosine kinase genes associate with KD (5, 6). One of the more consistent findings from GWAS data has been identifying SNPs in the region of the B lymphocyte kinase (BLK) (123, 125). BLK encodes a non-receptor protein tyrosine kinase and of the Src family of tyrosine kinases that acts downstream of the B cell receptor. Functional validation shows BLK is induced during acute KD (from patient samples) and the protective genotype of this SNP correlates with lower BLK expression on induction of EBV transformed B cells. Intriguingly, on a recent meta-analysis rs2736340 of BLK is also associated with systemic lupus erythematosus and rheumatoid arthritis-associated (126). Hypothesizing the importance of B cells based solely on BLK association is tenuous, as Blk knockout mice also suggest a role for Blk in the development of γδ TCR^+^ T cells (127) and marginal zone B cells (128). The association of CD40 would also potentially point to a more specific B cell function (5, 123, 125), although CD40 is expressed on a number of cell types and functional inference of this association is lacking. The association of BLK and FCGR2a were recently confirmed; however, did not seem to correlate with incomplete or cases from children older than 5 years of age (129).

A recent meta-analysis did see nominal associations with four previously noted genes (FCGR2A rs1801274, TCP1 rs3818298, BLK rs2736340, and CD40 rs4813003) but did not replicate a number of these associations (ZFHX3 rs9937546, NAALADL2 rs1870740, CAMK2D rs4834340, LNX1 rs6554112, MIA-RAB4B rs2233152, HLA-DQB-HLA-DOB rs2857151) (130). When specifically studying susceptibility to cardiac aneurysms, a number of intergenic associations were also shown, highlighting the complex organization and influence of chromatin structure (131). The most review and meta-analysis of available data supports a role for in susceptibility for genetic variations of ACE, BLK, CASP3, CD40, FCGR2A, FGb, HLA-E, IL1A, IL6, ITPKC, LTA, MPO, PD1, SMAD3, TARC/CCL17, and TNF. Also, genetic variations in BTNL2, CASP3, FCGR2A, FGF23, FGb, GRIN3A, HLA-E, IL10, ITPKC, and TGFBR2 may serve as biomarkers of CALs in KD (109).

Overall, association with a variety of pathways is not surprising for a disease with a likely complex pathogenesis. However, most of the major repetitively associated and/or validated genomic findings could support a role for B cells and antibodies influencing susceptibility (110).

### Clues From Treatment (See Table 2)

#### Intravenous Immunoglobulin (IVIG)

**Figure 1:**
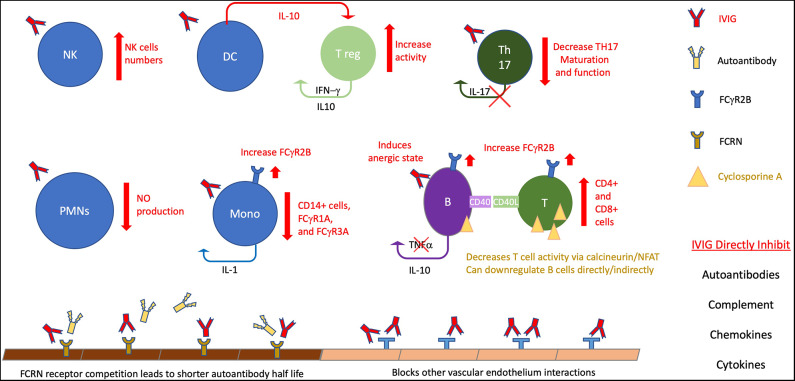
The many and varied potential activities in KD. This figure highlight potential therapeutic effects on B cell activity, particularly for IVIG. IVIG effects are noted by red elements, cyclosporin A noted by gold. B, B cells; T, T cells; T reg, T regulatory cells, DC, dendritic cells; nk, natural killer; PMNs, polymorphonuclear cells; Mono, monocytes, NO, Nitric Oxide.

The first implication of humoral immunity possibly being involved in KD was the response to immunoglobulin infusions. IVIG was started after success with preparations in Immune thrombocytopenic purpura and was shown to protect against cardiac involvement (142). After discovery that aneurysms are associated with KD, studies, including randomized controlled trials, supported a protective effect of IVIG (136). IVIG is known to broadly effect numerous mechanisms that have been outlined in recent reviews, including: direct neutralization of organism or toxin, inhibition of autoantibodies, inhibition of the complement cascade, inhibition of adhesion molecules on vascular endothelium, modulation of cytokine response, expansion of T regulatory cells, modulation of dendritic cell responses, and decreasing pro-inflammatory effects of monocytes by FC interaction (143–146). Possible mechanisms are detailed in Figure 1. The exact mechanism that correlates with clinical improvement seems to depend on the underlying disorder. On the most simplistic level, supplementation of a specific necessary immune response is still theorized for KD (147).

**Table 2:** Notable therapies and trials for treatment to prevent coronary aneurysms in Kawasaki Disease.

**Therapy**	**Mechanism and cohort**	**Clinical trials, National Clinical Trial (NCT) #,** **name (if applicable)**	**Results summary/comments**
Infliximab	TNFα blockade, most studied in refractory cases	2298062; 00271570; 00760435; 01596335; 03065244-KIDCARE	Given with IVIG, Improved defervescence, well-tolerated, variable z score reduction, (132). In refractory, Improved defervescence, well-tolerated, (133). KIDCARE recruiting
Etanercept	In conjunction with IVIG	00841789	Study awaiting results
Anakinra	IL-1 blockade, Refractory cases	0217985302390596-KAWAKINRA	KAWAKINRA recruiting
Cyclosporine A	In conjunction with IVIG in predicted high risk individuals	Japan Medical Association, JMA-IIA00174- KAICA	Efficacy shown in preventing more severe coronary involvement (134), (135)
IVIG dosage		00000520; 02439996	Single dose of IVIG is better than splitting doses (136)
IVIG + pulsed steroids	Primary cases	00132080	No difference shown (137)
IVIG + 5 days prednisolone	For refractory cases	03200561- RAST	Proposal published (138); recruiting
IVIG without Aspirin	Primary cases	02951234	Proposal published (139); recruiting
Doxycycline	Decreases MMP degradation of elastin, supported by murine model	01917721-DEAL	Proposal published (87); recruiting
Statins	For those with severe coronary artery involvement	03915795	Recruiting
Rituximab	Anti-CD20 targets B cells	na	Case report (140)
Plasma Exchange	Broad effects, decreases cytokines, increase of T regs	na	No active studies (141)

*na, not applicable*.

However, it is unclear how IVIG actually functions during KD and if specific antibody responses are responsible for pathogenesis. Early studies focused on dosing regimens and showed single large infusions (2 grams/kg) are superior to smaller more frequent doses (136). It is possible that a high level of antibody is needed to clear a pathogen, or to improve diffusion to certain areas; however, why this difference is unclear. The mechanism of loading of the neonatal FcRn receptor may require such high volume administration. Other Fc receptor modulation, particularly upregulation of FcγRIIB has also been proposed (148, 149). Without knowledge of the cause of KD, the mechanism of action will remain difficult to prove. The anti-inflammatory properties of IVIG also treat many infectious agents so a clinical response to IVIG is not definitive proof of the diagnosis of KD (147).

Recent studies show specific immune changes induced by treatment with IVIG. TH17 cells and IL-17 levels are elevated during acute KD, which is seen in autoimmune disorders, and this can be improved after IVIG (150, 151). T regulatory cells (T regs) are reduced during acute KD, and this is improved with IVIG (152). These cells, as well as myeloid dendritic cells are expanded by IVIG (145). In a study comparing IVIG to aspirin treatment alone group, IVIG treatment showed increase CD4, restoration of CD8 to normal control levels, and a significant suppression of CD19 cells below that of normal controls (153). Other studies also have explored diminished CD8 T cells in acute KD (154). Abnormal elevations of BAFF, which is in involved in survival of and proliferation of B cells, are decreased after treatment with IVIG (155). In what authors termed, “functional silencing,” IVIG suppressed phosphoinositide 3-kinase signaling (via NFAT) which would affect calcium signaling, coreceptor activation, and BCR activation (156). This finding supportive of B cell signaling involvement in KD could potentially link some of the genomic and transcriptomic associations.

#### Other Treatments

Adjunct steroids showed efficacy in certain populations (157). Meta-analysis of this and other clinical trials, mostly from Japan, support the use of steroids along with IVIG in high-risk patients (158). Since it is difficult to predict risk in heterogenous populations outside of Japan, and historical studies have shown worse outcomes with steroid use (159), there is not a universal recommendation to give adjunct steroids (2). Even in the revised 2012 guidelines from the Japanese Society of Pediatric Cardiology, “controversy remains” on universal initial steroid use (160). Aspirin is begun at anti-inflammatory doses initially, then is changed to a lower dose to prevent complications of coronary involvement (2). As this has not been shown to effect development of aneurysms, other platelet- aggregation inhibitors such as Clopidogrel or dipyridamole, can be considered in special situations (2). The main treatment modalities hoped to target prevention of coronary aneurysms used for refractory treatment are second doses of IVIG, steroids, calcineurin inhibitors, anti-IL-1 monoclonal antibodies, and anti-TNF monoclonal antibodies; all of which have broad immunological effects (53, 161).

Success with anti-TNFα treatment seemingly argues against a significant role of B cells, as this would affectively release a suppressive action of TNFα on B cell proliferation. The main treatments studied have been infliximab, a humanized mouse monoclonal antibody, and etanercept, a human recombinant mimic of the TNFα receptor (162). TNFα produced from B cells is implicated in atherosclerosis, so perhaps B cell derived TNFα is targeted (163). Notably, IVIG decreases TNFα production. The status of clinical trials have been recently reviewed (164). Although tolerance and improved defervescence are clear in trials with infliximab, there is less clarity of efficacy for improvement of aneurysms (132, 133). The KIDCARE trial, which is focused on recalcitrant cases may prove to be more insightful than previous trials. Etanercept has also undergone recent phase II randomized trial, but publications of results have not been done to date. A recent meta-analysis of twelve studies showed that both IV methylprednisolone and infliximab were not superior to a second dose of IVIG for prevention of aneurysms (165). The other main class of biologics used are Interluekin-1 (IL 1) inhibitors. These obviously have a broad inflammatory response (145) with notable effects on B cell activity (166) There is support in the *Lactobacillus casei* mouse model for IL-1 playing a role (167).

Genetic associations and data from murine models have supported targeting of NFAT with calcineurin inhibitors, and treatment protocols for KD have been developed (168). A recent Japanese clinical trial (135) show that addition of Cyclosporine A to IVIG in high risk patients is efficacious in prevention of the largest of aneurysms. Cyclosporine A mainly targets T cell activity by inhibition of calcineurin and its downstream transcription factor NFAT. This downregulation of T cell activity has been shown to decrease B cell immunoglobulin production (169). Although encouraging, the applicability of this treatment outside of the highest risk individuals is unclear. Limited reports of successful treatment with anti-B cell monoclonal antibodies (anti-CD20) also support a role for B cell activation in KD pathogenesis (140). Based on data regarding MMP in murine models, doxycycline is also being used (87). Supplementation with statins also has a currently recruiting trial ongoing. Plasma exchange, which also has a broad range of activity, is used in some centers, particularly in Japan (2). Recent data suggests restitution of circulating T regulatory cells occurs which aligns with other mechanisms similar to IVIG (141).

The broad effects of most treatments make drawing specific conclusions about correlation with pathogenesis (see Figure 1); however, a number of treatments and reported success with anti-CD20 monoclonal antibodies supports a significant role for B cells.

### Autoimmune Antibodies in KD

A role for B cells could imply a significant autoimmune component in KD (170). Self-antigen responses to a variety of targets have actually been well-described in KD. These include recent reports of antibody responses to type III collagen, myosin (171), cardiolipin (172), alpha-enolase (173), and anti-endothelial antibodies. Anti-endothelial antibodies are particularly interesting as these are seen in other disorders, such as SLE and renal allograft rejection (174). Other vasculitides have also been associated with anti-endothelial antibodies. These have been shown to cause upregulation of E-selectin, VCAM-1, ICAM-1, and NFκB (175). Responses to these antibodies include upregulation of inflammatory cytokines and apoptosis of the endothelial cells.

In KD subjects, a polyclonal response against endothelial cells has been described (176); however, not universally (177). Particularly during generalized inflammation, cytokines such as IFN-γ, IL-1, and TNF can reveal this IgG and IgM anti-endothelial response (178, 179). In cell lysis assays, pathogenesis was eliminated by clearing the serum through anti-IgG and anti-IgM sepharose columns arguing against a role of peripheral anti-IgA responses. This does not eliminate the potential role of intra-tissue IgA+ plasma cell development in pathogenesis as has been postulated (180, 181). Other studies support significant IgM mediated cytotoxicity against endothelial cells in KD patients (182). Prevalent IgM anti-endothelial responses in KD have also been shown without cytokine stimulation (182, 183). In a mouse model system, anti-endothelial antibody responses were replicated, but these did not demonstrate cardiac vascular involvement (184).

Recently, the autoimmune considerations in KD have been reviewed (170). The case report of anti-B cell monoclonal antibody success was proposed by the authors to be due to the downregulation of such an anti-endothelial invasive effect (140).

Although intriguing, it remains unknown if these anti-endothelial responses actually contribute to the vasculitis in KD and other vasculitides (175).

### Human Pathologic Studies

A small number of studies, due to the necessary reliance on autopsy specimens, support limited B cell infiltration into the coronary arteries (185). These limited studies have shown that coronary infiltrates develop over time with late fibrosis occurring in the intima and adventitial layers and can be divided into an acute, subacute and chronic state formation (90). In the acute phase, neutrophils are the predominant initial cell infiltrate (186). This can result in necrotizing arteritis that destroys the adventitia leading to aneurysm formation (90).

A number of studies have noted lymphocytic infiltrates in samples from later timepoints. In a study of eight specimens, two of which were within the first 2 weeks of illness, showed predominant monocytes on day 6 and no CD3+ (T cell) nor CD20+ (B cells) cells. T and B cells were shown in the one sample from day 10 but both monocytes and neutrophils were more predominant (186). B cell responses were highest on day 10 and 17 overall, but one of the two samples from day 17 had very few B cells. Another small study of seven subjects, all from over 13 days of illness, showed IgA+ plasma cell infiltrates. These were seemingly specific to KD; however, the fourteen control specimens were from autopsies that succumbed generally from non-inflammatory and non-cardiac syndromes. Notably, mature memory and immature B cells (CD20+ cells) were lacking (180). If the previous day 10 B cell infiltrates became plasma cells as has been postulated (187), these studies would be consistent. Also consistent, in a series of six KD specimens who were not diagnosed with aneurysms compared to 21 non-inflamed controls, neutrophil infiltration in adventia and intima layers was quickly followed by lymphocyte infiltrates, then mixed lymphocyte, eosinophils, and plasma cell infiltrates were demonstrated later, near day 19 of illness (186, 188). Other reports support monocyte infiltration with more significant CD8 T cell responses, however there were few early samples here as well (187). Overall, innate immune cells are the predominant in early infiltrates.

Prominent nodular infiltrates, similar to atherosclerotic plaque formation, have also been described, but these appear to occur at later timepoints (>3 weeks). These infiltrates consisted of T cells, macrophages, B cells and prevalent IgM+ plasma cells, with less frequent IgA+ plasma cells. The authors compare these to similar B cell rich lesions driven by both superantigens and specific infectious antigens (189).

The largest study, relying on electron microscopic studies, suggests that there is an early necrotizing arteritis suggestive of an acute viral infection, followed by a vasculitis, then luminal myofibroblast proliferation (90). There were a number of differences from previous reported pathological specimens, including lack of granulomatous inflammation, lack of small vessel pathology, minimal medial hyperplasia or scarring, and lack of atherosclerosis. Overall most studies are consistent with an early phase with neutrophil infiltration when severe is termed necrotizing arteritis, with later lymphocyte and plasma cell infiltration still in the background of significant monocytes.

Only few studies on long-term sequelae have been published. There seems to be an increased risk of cardiac death in survivors of KD. Limited human pathology specimens show no long-term inflammatory infiltrates (186, 188). The limited data on adult follow-up cases of KD implies there is a greater lifetime risk of cardiac issues and early mortality (15–18). Even those who had KD without noticeable aneurysms may have endothelial dysfunction and increased arterial stiffness (158). A clinical trial on long-term consequences is actively recruiting (NCT03750123-CAVASAKI trial). As established clinical cohorts age, the lifetime risk of childhood KD should be revealed.

Unfortunately, disparity of reports, lack of early timepoints, and lack of control tissue sample from cases experiencing ongoing inflammation make firm conclusions difficult. Collection and study of these types rare samples should continue.

### Studies on Circulating Immune Cells

In the few published studies on peripheral cell subsets during acute KD, circulating B cells are generally elevated (6, 9, 45, 71, 190). Lack of changes in acute and convalescent B cells subgroups and increases in CD69+ natural killer and γδ T cells supported a role for the innate immune system (71). B cells did seem to be in an “activated” phenotype, being positive for CD86 (190). After stimulation of the TLR-9 receptor there was a global increase in the ability of B cells to secrete IgM, IgG, and IgA with a notable expansion in IgA+ B cell numbers (190). One study showed a paucity of IgA + peripheral B cells from acute KD samples compared to controls continuing through convalescence (8) that was not replicated in other studies. Reports do show increased IgA immune complexes and levels (13), although immune complexes do not necessarily portend worse prognosis (191). Overall, in the small number of studies relating to the peripheral blood B cell compartment, overall B cells numbers are increased and B cells are more reactive.

Although, total numbers of cells do not show consistent results, clonal expansion within the B cell compartment can be studied. A specific immune response to an agent typically has an initial inherent immune component that leads to antigen presentation to effector cells. Receptors on the effector cell surface (T-cell receptors in T cells and Immunoglobulin, or antibody, in B cells) bind specific targeted areas of the agent, termed epitopes. Specific recognition by T and B lymphocytes leads to stimulation, lymphocyte replication and clonal expansion; what is termed an oligoclonal response. Oligoclonal expansion is shown in peripheral IgM+ B cells in KD (9). Oligoclonal plasma cell infiltrates, predominantly IgA+, have been shown in KD arterial specimens (192). Heavy and light chain sequences were obtained and cloned into full length expression vectors from these cardiac vessel samples. These chimeric antibodies identified intracellular inclusions (ICI) in bronchial epithelium samples from children with KD (46, 104). However, similarly cloned antibodies revealed self-antigen targeting (181). Additionally, 26% of the control group, composed primarily of adult patients, had similar inclusion bodies, and next generation sequencing from these ICIs did not reveal an etiology (193). Although plasma cell infiltration outlined above is intriguing, a similar pathological response is seen in a number of inflammatory conditions such as NMDAR encephalitis (194), primary sclerosing cholangitis, (195) multiple sclerosis, (196) and responses to tumors (197). ICI in cells can be any number of structures (aggresomes, stress granules, p-bodies, prion-aggregates, aggresome-like induced structures (ALIS) and autophagosomes) and this has been recently reviewed (164). Currently, it is unclear of the significance of this work.

### Similar Plasmablast (PB) Responses in KD Compared to Infections

Plasmablasts (PBs), derived from naïve and memory responses, are B cells transitioning to plasma cells that circulate in the peripheral blood cell compartment. They are characterized by surface expression of CD19, with CD20 downregulation, and high levels of CD27 and CD38. After antigenic challenge (vaccination and natural infections), CD19+, CD20lo, CD27+ and CD38 + PBs can be seen in the peripheral blood (8, 198–200). Immunization studies in adults, with pathogens such as tetanus, influenza and rabies, show PBs are enriched for specific antibodies against the challenge antigen, temporally peak 5–10 days after immunization, and are predictive of later sero-immunity (198, 201–203). In comparison to the general circulating B cell population, PBs are enriched for B cells that produce infection-specific antibodies (204–209) Although certain infections, such as dengue virus, may set off exceedingly high PB levels (210–212), excessive PB responses are usually an indicator of autoimmune flares (213). This excessive circulating PB response seen specifically correlates with CRP level in studies on ulcerative colitis (212, 214) and IGG4 related disease (215, 216). Besides correlation of an inflammatory state consistent with infection and inflammation, little is known regarding regulatory or pathologic consequences of PB elevation.

We postulated if KD is caused by an infection, we should see a predictable rise of PBs in the peripheral blood. On single timepoint collection, we showed 15 of 18 KD samples had elevation of their PBs, and overall this response was similar to the range of data shown in our 69 infected controls (45). Results of this study are consistent with the majority of the literature that show B cell stimulation and increasing peripheral B cell numbers during KD (6, 9, 71, 190). Importantly, the levels were not consistent with a pure autoimmune response as they did not correlate with CRPs and were not overly excessive. Unfortunately, only five children had repeat samples, but from these it did not appear that IVIG has an effect on PB numbers. As PBs rise and fall over time, we have ongoing studies collecting samples over time to confirm a dynamic PB response similar to infections occurs in KD. Other groups have recently shown similar PB elevation during KD, but this was compared to healthy controls (217).

Ongoing studies are exploring heavy and light chain usage in B cells and PBs during KD with next generation sequencing techniques. From our initial evaluation, a number of these clones have markers of affinity maturation (multiple clonal members, isotype switching, increased nucleotide substitutions from predicted germlines, and increases in the subgroup replacement to silent nucleotide mutation (R/S) ratios) (218). Our group and other are expressing cloned sequences as monoclonal antibodies in the attempt to find their antigenic target.

Because of reported oligoclonal responses and characteristics suggesting response to infection seen in PBs and antibodies during KD, we hypothesize the antibodies derived from PBs during KD are specific against the etiology that led to KD within that child.

## Discussion

As reviewed, a number of studies support a roll for B cells, plasma cells, and antibodies in the pathogenesis of KD. Although B cell and plasma cell infiltration in pathology specimens is intriguing, whether they are bystanders activated by a superantigen effect, are responding to a self-antigen revealed by inflammation, or specific against an infectious etiology is currently unknown. An autoimmune component to this disease has long been postulated, and overlapping genomic risk associations with lupus and rheumatoid arthritis suggest this to play a role. Genomic and proteomic approaches strongly suggest a role for the innate immune system in pathogenesis, but associations with BLK, and CD40 also support a potential role for B cells. The increasingly appreciated role of B cells as immune system modulators may explain these associations and align the disparate data.

Humoral immune responses continue to be worth exploration in children with KD. Like the mouse models and attempts at developing new therapeutics, it is hard to be confident in any one approach without knowledge of the etiology. Compelling recent data suggests that exploration of the specific B cell responses is an encouraging path to discovering improved diagnostics and potentially the pathogen that sets off this immune cascade.

This is a rich opportunity for clinical investigators. Rigorous studies are needed on those children who present with KD. If any pulmonary findings are found, bronchial washings should be obtained and stored for potential molecular diagnostics. Other samples, such as PBMCs and serum, should be taken and banked for future studies. Thorough autopsy evaluation should be pursued on any subjects who succumb during the acute or convalescent phases of KD. Improved reporting and national registries would go a long way in establishing a representative pool of patients. Studies currently ongoing on peripheral cytokine profiles, B cells and PBs may show a consistent marker to help define who has KD. A correlative diagnostic marker, possibly even antibody derived, would be a highly desirable first step in future studies.

As outlined herein, evidence from genomics, transcriptomics, and proteomics support a role for B cells in the pathogenesis of KD, and continued studies on B cell responses may assist in identifying the etiology of KD.

## Author Contributions

MH conceived and was the sole contributor to this manuscript.

## Conflict of Interest

The author declares that the research was conducted in the absence of any commercial or financial relationships that could be construed as a potential conflict of interest.
